# Supplementary material for: Top Canopy Height and Stem Size Variation Enhance Aboveground Biomass across Spatial Scales in Seasonal Tropical Forests
Source: Plants (Basel). 2023 Mar 16;12(6):1343. doi: 10.3390/plants12061343 (PMC10051130; doi:10.3390/plants12061343)

Appendix A: Supplementary information

Table S1. Summary of the variables used in the study

Variable	Unit	Mean	S.D.	Range
**Small scale, 20 m × 20 m scale, 1000 quadrats**				
Elevation	m	818	117	659-1049
TCH (Mean top canopy height)	m	23.4	7.4	6.6-49.7
Stem size variation	unitless	1.42	0.36	0.47-3.39
Stem abundance	N ha^{-1}	4125	1375	450-8800
Species richness	unitless	43	9	10-78
AGB (Aboveground biomass)	Mg ha^{-1}	317.9	224.8	30.4－1557
**Large scale, 50 m × 50 m scale, 160 quadrats**				
Elevation	unitless	818	117	659-1049
TCH (Mean top canopy height)	m	23.4	6.0	12.1-42.9
Stem size variation	unitless	1.54	0.24	0.92-2.22
Stem abundance	N ha^{-1}	4244	1116	1956-7380
Species richness	unitless	107	18	68-149
AGB (Aboveground biomass)	Mg ha^{-1}	262.4	127.9	48.9-719.1

Note: original variables are shown here, whereas transformed datasets are used in analysis.

Table S2. Direct, indirect and total standardized effects of predictors on AGB (aboveground biomass) at scale of 20 m × 20 m, based on the structural equation model. Significant effects (P < 0.05) are indicated in bold. Abbreviations are explained in Table S1. See model in Fig. 2a

**Response variable**	**Mediator variable**	**Predictor variable**	**Effect**	**Beta**	**S.E.**	***Z-value***	***P-value***
AGB		Elevation	Direct	0.10	0.02	5.95	**0.000**
AGB		TCH	Direct	0.21	0.02	10.43	**0.000**
AGB		Stem abundance	Direct	0.17	0.02	8.17	**0.000**
AGB		Stem size variation	Direct	0.71	0.02	35.00	**0.000**
AGB		Species richness	Direct	0.04	0.02	1.90	0.057
TCH		Elevation	Direct	-0.22	0.03	-7.11	**0.000**
Stem abundance		Elevation	Direct	0.20	0.03	6.49	**0.000**
Stem size variation		Elevation	Direct	-0.05	0.03	-1.77	0.077
Species richness		Elevation	Direct	0.03	0.03	1.18	0.237
Stem size variation		TCH	Direct	0.62	0.03	24.68	**0.000**
Species richness		TCH	Direct	0.04	0.03	1.28	0.200
Stem size variation		Stem abundance	Direct	-0.03	0.03	-1.29	0.199
Species richness		Stem size variation	Direct	0.09	0.03	3.02	**0.002**
AGB	TCH	Elevation	Indirect	-0.05	0.01	-5.88	**0.000**
AGB	Stem abundance	Elevation	Indirect	0.04	0.01	5.08	**0.000**
AGB	Stem size variation	Elevation	Indirect	-0.03	0.02	-1.76	0.078
AGB	Species richness	Elevation	Indirect	0.00	0.00	1.00	0.315
AGB	Stem size variation	TCH	Indirect	0.44	0.02	20.17	**0.000**
AGB	species richness	TCH	Indirect	0.00	0.00	1.06	0.288
AGB	Stem size variation	Stem abundance	Indirect	0.00	0.00	-1.06	0.287
AGB	Species richness	Stem abundance	Indirect	0.03	0.01	1.90	0.058
AGB	Species richness	Stem size variation	Indirect	0.00	0.00	1.61	0.108
AGB	TCH<br>Species richness<br>Stem abundance<br>Stem size variation	Elevation	Indirect	-0.04	0.02	-2.06	**0.039**
AGB	Species richness<br>Stem size variation	TCH	Indirect	0.44	0.02	20.24	**0.000**
AGB	Species richness<br>Stem size variation	Stem abundance	Indirect	0.00	0.02	0.15	0.881
AGB	Species richness	Stem size variation	Indirect	0.00	0.00	1.61	0.108
AGB	TCH<br>Stem abundance<br>Stem size variation<br>Species richness	Elevation	Total	0.06	0.03	2.12	**0.034**
AGB	Stem abundance<br>Stem size variation	TCH	Total	0.66	0.02	27.20	**0.000**

	Species richness						
AGB	Species richness<br>Stem size variation	Stem abundance	Total	0.18	0.02	7.36	**0.000**
AGB	Species richness	Stem size variation	Total	0.72	0.02	35.28	**0.000**
Species richness	TCH<br>Stem abundance<br>Stem size variation	Elevation	Total	0.15	0.03	4.71	**0.000**
Species richness	Stem size variation	TCH	Total	0.10	0.02	3.97	**0.000**
Stem size variation	TCH	Elevation	Total	-0.19	0.03	-6.04	**0.000**

Table S3. Direct, indirect and total standardized effects of predictors on AGB (aboveground biomass) at scale of 50 m × 50 m, based on the structural equation model. Significant effects (P < 0.05) are indicated in bold. Abbreviations are explained in Table S1. See model in Fig. 3a,

**Response variable**	**Mediator variable**	**Predictor variable**	**Effect**	**Beta**	**S.E.**	***Z-value***	***P-value***
AGB	--	Elevation	Direct	0.12	0.04	3.23	**0.001**
AGB	--	TCH	Direct	0.55	0.06	9.79	**0.000**
AGB	--	Stem abundance	Direct	0.30	0.04	6.64	**0.000**
AGB	--	Stem size variation	Direct	0.44	0.06	7.74	**0.000**
AGB	--	Species richness	Direct	-0.11	0.04	-2.58	**0.010**
TCH	--	Elevation	Direct	-0.29	0.08	-3.84	**0.000**
Stem abundance	--	Elevation	Direct	0.20	0.08	2.59	**0.010**
Stem size variation	--	Elevation	Direct	-0.05	0.05	-0.95	0.342
Species richness	--	Elevation	Direct	-0.03	0.07	-0.51	0.614
Stem size variation	--	TCH	Direct	0.75	0.05	14.84	**0.000**
Species richness	--	TCH	Direct	-0.04	0.10	-0.40	0.691
Stem size variation	--	Stem abundance	Direct	-0.11	0.05	-2.29	**0.022**
Species richness	--	Stem size variation	Direct	-0.07	0.10	-0.66	0.507
AGB	TCH	Elevation	Indirect	-0.16	0.04	-3.58	**0.000**
AGB	Stem abundance	Elevation	Indirect	0.06	0.02	2.41	**0.016**
AGB	Stem size variation	Elevation	Indirect	-0.02	0.02	-0.94	0.346
AGB	Species richness	Elevation	Indirect	0.00	0.01	0.50	0.620
AGB	Stem size variation	TCH	Indirect	0.33	0.05	6.86	**0.000**
AGB	species richness	TCH	Indirect	0.01	0.01	0.39	0.695
AGB	Stem size variation	Stem abundance	Indirect	0.01	0.01	1.71	0.087
AGB	Species richness	Stem abundance	Indirect	-0.07	0.03	-2.49	0.013
AGB	Species richness	Stem size variation	Indirect	0.01	0.01	0.64	0.521
AGB	TCH<br>Species richness<br>Stem abundance<br>Stem size variation	Elevation	Indirect	-0.12	0.06	-2.10	**0.036**
AGB	Species richness<br>Stem size variation	TCH	Indirect	0.33	0.05	6.77	**0.000**
AGB	Species richness<br>Stem size variation	Stem abundance	Indirect	-0.12	0.04	-3.34	**0.001**
AGB	Species richness	Stem size variation	Indirect	0.01	0.01	0.64	0.521
AGB	TCH<br>Stem abundance<br>Stem size variation<br>Species richness	Elevation	Total	0.00	0.07	0.03	0.976
AGB	Stem abundance,<br>Stem size variation	TCH	Total	0.88	0.04	20.00	**0.000**

	Species richness						
AGB	Species richness<br>Stem size variation	Stem abundance	Total	0.18	0.04	4.23	**0.000**
AGB	Species richness	Stem size variation	Total	0.45	0.06	7.73	**0.000**
Species richness	TCH<br>Stem abundance<br>Stem size variation	Elevation	Total	0.10	0.08	1.24	0.217
Species richness	Stem size variation	TCH	Total	-0.09	0.07	-1.39	0.165
Stem size variation	TCH	Elevation	Total	-0.29	0.08	-3.86	**0.000**

Fig. S1. Pearson's correlation coefficient between all pairs of variables at scale used in this meta-analysis. Red to green color indicates negative to positive correlation, the shade extents of circles represent correlation coefficient (*r*) size, and insignificant correlations ($P > 0.05$) include crosses inside the circles. Abbreviations for variables are explained in Table S1.

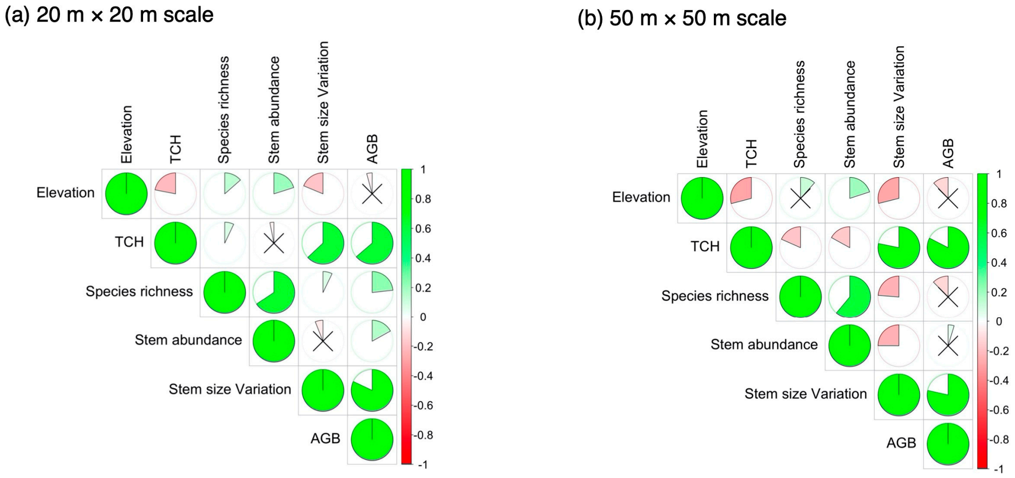

Supplement: Supplementary file 1 [file plants-12-01343-s001.zip › plants-2112947-supplementary.pdf]
